# MRI-Based Synovial Iron Quantification Associates with Bone Erosion in Rheumatoid Arthritis

**DOI:** 10.3390/biomedicines14040749

**Published:** 2026-03-25

**Authors:** Shuyuan Zhong, Churong Lin, Jianhua Ren, Yuhang Li, Bo Dong, Weihang Zhu, Yutong Jiang, Zetao Liao, Yanli Zhang, Liudan Tu, Minjing Zhao, Dongfang Lin, Ke Hu, Chenyang Lu, Yunfeng Pan, Yan Liu

**Affiliations:** 1 Department of Rheumatology and Immunology, Third Affiliated Hospital of Sun Yat-sen University, Guangzhou 510630, China; zhongshy27@mail2.sysu.edu.cn (S.Z.); dongb27@mail2.sysu.edu.cn (B.D.); zhuwh33@mail2.sysu.edu.cn (W.Z.); jiangyt7@mail.sysu.edu.cn (Y.J.); liaozt@mail.sysu.edu.cn (Z.L.); zhyanli5@mail.sysu.edu.cn (Y.Z.); tuliudan3@mail.sysu.edu.cn (L.T.); zhaomj3@sysu.edu.cn (M.Z.); lindongf@mail.sysu.edu.cn (D.L.); huke0506@163.com (K.H.); 2 Department of Radiology, Third Affiliated Hospital of Sun Yat-sen University, Guangzhou 510630, China; linchr6@mail.sysu.edu.cn; 3 Department of Joint and Trauma Surgery, Third Affiliated Hospital of Sun Yat-sen University, Guangzhou 510630, China; renjh@mail2.sysu.edu.cn (J.R.); liyh383@mail.sysu.edu.cn (Y.L.)

**Keywords:** synovial iron, bone erosion, magnetic resonance imaging (MRI), rheumatoid arthritis, quantification, risk factors, knee

## Abstract

**Objective**: To evaluate the utility of synovial iron quantification using Magnetic resonance imaging (MRI) in assessing structural joint damage in the knee of patients with rheumatoid arthritis (RA). **Methods**: This cross-sectional study employed a two-stage design. In the initial comparative stage, 6 patients with RA and 5 patients with osteoarthritis (OA) were recruited to compare synovial R2* values, a metric derived from iterative decomposition of water and fat with echo asymmetry and least-squares estimation quantitation (IDEAL-IQ) MRI sequences representing synovial iron content. Following this, the RA cohort was expanded to a total of 51 patients to investigate the association between R2* values and clinical parameters, including disease activity and bone erosion. Synovial fluid iron levels were measured with an Iron Assay Kit and synovial iron deposits were semi-quantified via Prussian blue staining. Associations between R2* and clinical and laboratory parameters, including inflammatory factors and joint damage indices, were analyzed using Spearman’s rank correlation. Univariate and multivariate ordered logistic regression models were employed to identify factors associated with bone erosion severity. An R2*-based nomogram was developed and validated using receiver operating characteristic (ROC) analysis and calibration curves. **Results**: Synovial R2* values were significantly higher in RA patients than those with osteoarthritis (53.66 S^−1^ vs. 31.38 S^−1^, *p* < 0.05), consistent with Prussian blue staining results. While synovial R2* values showed no significant correlation with systemic iron metabolic markers, inflammatory indicators, or the Disease Activity Score 28, they were positively correlated with bone erosion severity (ρ = 0.500, *p* < 0.001) and negatively associated with the joint space width (ρ = −0.307, *p* < 0.05). Multivariate analysis identified R2* as an independent indicator linked to bone erosion extent (OR = 2358.336, *p* < 0.001). The R2*-based nomogram demonstrated good discriminative performance. (AUC = 0.83). **Conclusions**: The R2* value derived from IDEAL-IQ MRI is a reliable tool for quantifying synovial iron and may represent a promising non-invasive imaging biomarker reflecting bone erosion in RA patients.

## 1. Introduction

Rheumatoid arthritis (RA) is a common chronic inflammatory disease with features of autoimmunity, characterized by joint pain, swelling, and deformities. The primary pathological features include synovial hyperplasia and bone destruction [1]. Previous studies have indicated that radiographic bone damage can progress even during clinical remission [2], suggesting the independence of joint pathology from systemic circulatory indicators. Microenvironmental disruptions, such as glycolysis dysregulation, oxidative stress, and lactate accumulation, have been observed in RA-affected joints [3,4,5]. These findings underscore the necessity for a deeper investigation into joint-specific pathogenesis to identify novel biomarkers and therapeutic targets.

Iron is an essential trace element critical for mitochondrial respiration, gene regulation, enzyme production, and DNA repair. However, excess iron can be toxic, generating oxygen radicals through Fenton chemistry, leading to cellular and tissue damage [6]. Iron overload is implicated in various diseases, including neurodegenerative diseases (e.g., Alzheimer’s disease and Parkinson’s disease), metabolic conditions (e.g., type 2 diabetes and obesity), musculoskeletal disorders (e.g., hemochromatosis arthropathy) and inflammatory diseases (e.g., colitis) [7,8,9,10]. Previous studies have reported increased iron accumulation in synovial tissues and synovial fluid from RA patients, compared to individuals with osteoarthritis (OA), hemophilic arthropathy, as well as healthy controls [11,12,13]. Excess iron may trigger ferroptosis, an iron-dependent form of regulated cell death characterized by lipid peroxidation, potentially exacerbating synovial inflammation in RA [11]. In contrast, chronic inflammation can lead to anemia of inflammation, a condition in which interleukin-6 (IL-6) stimulates hepcidin production, thereby reducing circulating iron levels. This type of anaemia occurs in approximately 64% of patients with RA [14,15]. However, the impact of exogenous iron supplementation in rheumatoid arthritis remains controversial. Some clinical data indicate that intravenous iron therapy can safely correct anemia in RA patients without evidence of disease flare or increased activity during follow-up [16]. In contrast, other reports have shown that administration of iron dextrose for anemia can exacerbate synovitis in RA within 24–48 h post-injection [17]. These conflicting observations suggest that while systemic iron supplementation may be safe in some contexts, localized iron deposition could potentially contribute to disease activity.

Atomic absorption spectroscopy, chemical colorimetry, and Perl’s Prussian blue staining are classic methods for iron assessment, with the latter two commonly applied to RA synovial fluid and synovium [13,18]. However, these methods require tissue or fluid specimens, making them invasive and subject to limitations such as sampling variability and low repeatability, which hinder their broader clinical application. Magnetic resonance imaging (MRI), a widely used radiological tool in clinical practice, offers a non-invasive alternative for iron detection. MRI is highly sensitive to iron, as excess iron increases both spin-echo (SE) R2 and gradient echo (GRE)-based R2* relaxation rates. These changes can be quantified using the iterative decomposition of water and fat with echo asymmetry and least-squares estimation quantitation (IDEAL-IQ) sequence, which has great advantages such as fast scanning times and no requirement for specialized post-processing [19,20]. Currently, MRI-based iron quantification is extensively used in conditions affecting the liver and brain [21,22,23]. The R2* parameter, in particular, is recognized as a reliable measure of liver iron concentration (LIC) by guidelines from the European Society of Gastrointestinal and Abdominal Radiology (ESGAR) and the Society for Abdominal Radiology (SAR) guideline recommendations [19]. Additionally, MRI plays a pivotal role in evaluating RA-related inflammation and structural damage through the RA MRI scoring system (RAMRIS), which evaluates bone erosion, joint space width, bone marrow edema (BME), and synovitis [24]. Despite these advancements, the application of MRI to evaluate synovial iron in RA patients has not yet been explored.

In this study, we utilized MRI as a non-invasive technique to assess iron accumulation in the knee joints and employed the R2* relaxation rate to quantify synovial iron content in a clinical cohort of RA patients. To validate the reliability of MRI in quantifying synovial iron, we compared the R2* values with results from traditional iron assessment methods, examining the consistency of iron quantification trends between RA patients and the control group. Additionally, we investigated the relationship between synovial R2* values and disease activity, as well as knee joint pathologies including synovitis, BME, joint space, and bone erosion, aiming to highlight the value of MRI-detected iron overload in RA progression and its potential implications for clinical assessment and management.

## 2. Materials and Methods

### 2.1. Patient Enrollment and Study Design

**Figure 1:**
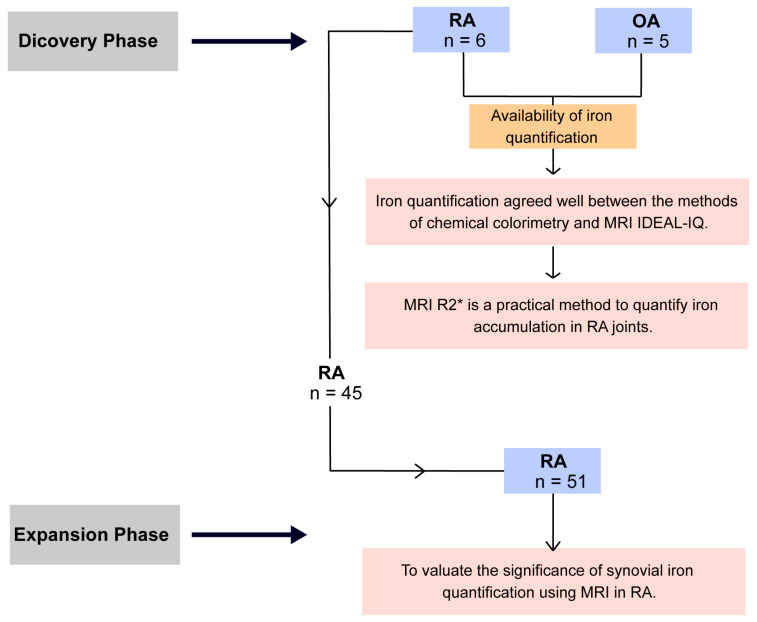
Flowchart of study subjects completing MRI of knee joints after applying exclusion criteria. RA, *n* = 51; OA, *n* = 5. RA: rheumatoid arthritis; OA: osteoarthritis.

Between July 2022 and December 2023, we consecutively enrolled patients from our department who presented with unilateral or bilateral knee swelling or pain and met the diagnostic criteria for RA or OA. Patients with RA were diagnosed according to the 1987 ACR criteria or the 2010 ACR/EULAR criteria, and were required to have no radiological or clinical evidence of knee osteoarthritis (e.g., osteophytes). Patients with OA were diagnosed based on the 1986 ACR criteria and served as controls (Figure 1). Participants with a history of previous knee dysplasia, metabolic bone diseases, tumors, septic arthritis, other infectious pathologies, other autoimmune diseases (e.g., reactive arthritis, psoriatic arthritis, etc.), or systemic iron overload disorders (e.g., hemochromatosis) were excluded. Patients with contraindications to MRI scanning were also excluded. In addition, individuals who had received iron therapy or been prescribed medications affecting iron metabolism within the preceding six months were excluded from the study. All study participants underwent a comprehensive evaluation by two rheumatologists with over 10 years of clinical experience. This study was approved by the Medical Ethics Committee (RG-2023-080-01) and conducted in accordance with the Declaration of Helsinki. All participants provided written informed consent.

This study was conducted in two sequential phases. In Phase 1 (discovery phase), an initial cohort of patients with RA (*n* = 6) and OA (*n* = 5) was utilized to assess the feasibility of R2* measurement for iron quantification and its consistency with synovial pathology. In Phase 2 (expansion phase), the RA cohort was expanded to a total of 51 patients (including the initial 6 RA cases). This larger group served to investigate the association between MRI-derived iron levels and markers of disease progression.

### 2.2. Data Collection

The evaluation included demographic and clinical features, including age, gender, height, weight, BMI, and disease duration. A comprehensive analysis of laboratory parameters was also performed, including C-reactive protein (CRP), erythrocyte sedimentation rate (ESR), serum iron, serum ferritin, transferrin saturation, rheumatoid factor (RF), anti-citrullinated protein antibodies (ACPA), serum phosphorus, serum calcium and 25 hydroxyvitamin D3. The clinical assessment included the tender joint counts (TJC), swollen joint counts (SJC), visual analogue scale for pain (VAS, ranging from 0 to 10), disease activity score in 28 joints (DAS28), health assessment questionnaire (HAQ, ranging from 0 to 3) and medication history. All patients were assessed by two experienced rheumatologists independently.

### 2.3. Iron Quantification in Synovial Fluid and Synovium

The synovial fluid (SF) specimens from 16 RA patients and 14 OA patients (as controls) were collected, and iron concentration was measured with an Iron Assay Kit (NjjcBio, Nanjing, China, A039) according to the manufacturer’s instructions. For iron detection, synovial tissues were collected from 10 RA and 10 OA patients undergoing total knee arthroplasty or arthroscopic surgery. These tissues were fixed with 4% paraformaldehyde, embedded in paraffin and then sectioned into 4 μm thick slices. Prussian blue staining (Servicebio, Wuhan, China, G1029) was performed to assess the deposited iron and quantified using the ImageJ software (v 1.52a, Washington, DC, USA).

### 2.4. MRI Acquisition

Fifty-one RA and five OA patients underwent MRI scanning of their knee joints in the supine position using a 3.0 T MR scanner (MR750; GE Healthcare, Milwaukee, WI, USA). The conventional MRI examination included sagittal, axial and coronal T1-weighted and T2-weighted fat suppression (T2-FS) images before contrast administration (gadolinium contrast agent), as well as T1-weighted fat suppression contrast-enhanced images (T1 FS + C). In addition, the sagittal R2* images in IDEAL-IQ sequence were performed to analyze iron deposition in the knee joints. Detailed scan parameters of each MRI sequence are provided in Supplementary Table S1.

**Figure 2:**
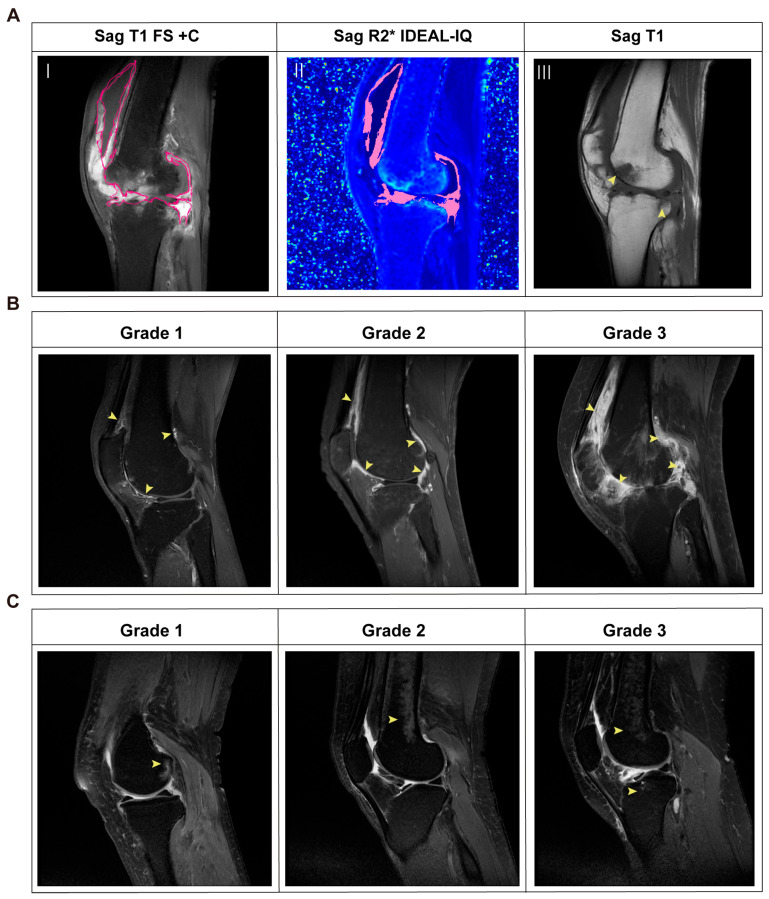
Assessment of synovial iron deposition and associated structural joint damage based on MRI. (**A**) MRI images from a patient used for evaluation of synovial iron and bone erosion. (I) Synovitis on sagittal T1 FS + C images (outlined in pink). (II) Iron examination in the synovium on sagittal R2* IDEAL-IQ images (outlined in pink). (III) Bone erosion (shown in yellow arrows) on sagittal T1 images. (**B**) Grading of synovitis on T1 FS + C images. (**C**) Grading of BME on T2-FS images. Grade 1 = mild, Grade 2 = moderate, Grade 3 =severe. Sag, sagittal; BME, bone marrow edema; T1 FS + C, T1-weighted fat suppression contrast-enhanced; IDEAL-IQ, Iterative Decomposition of Water and Fat with Echo Asymmetry and Least-Squares Estimation; T2-FS, T2-weighted fat suppression.

All MRI parameter evaluations were conducted using the ITK-SNAP 3.5 Workstation. Three consecutive sagittal slices were employed to highlight the overall synovium region of interest (ROI), centered on the layer showing the maximal representation of the femorotibial joint, hypertrophied synovium and joint space. The synovial ROI was manually delineated on T1-weighted fat-saturated contrast-enhanced (T1 FS + C) images to maximize inclusion of synovium while strictly excluding bone, fat, and joint effusion. After ROI delineation, the workstation calculated the mean synovial volume and automatically derived the corresponding mean R2* values (S^−1^) from the IDEAL-IQ R2* sequence, providing an indirect quantification of synovial iron deposition (Figure 2A). All inflammation, bones and joints were scored semi-quantitatively according to the RAMRIS method (20). BME was assessed on T2-FS images using a 0–3 scale based on the proportion of affected bone: 0 = none; 1 = mild (1–33%); 2 = moderate (34–66%); 3 = severe (67–100%) (Figure 2C). Synovitis was evaluated on T1-FS + C images using the same scale, based on the proportion of enhancing tissue in the synovial compartment relative to the presumed maximum volume (Figure 2B). On T1-weighted images, bone erosions were graded on a scale from 0 to 10, based on the proportion of eroded bone relative to the “assessed bone volume,” which extends from the articular surface to a depth of 1 cm. Erosion severity was scored as follows: 0 = no erosion, 1 = 1–10% of bone eroded, 2 = 11–20%, and so on (Figure 2A). Joint space width (JSW) was quantified by averaging the minimum vertical distance between the femoral condyle and tibial plateau across three consecutive coronal T2-FS slices (the narrowest slice and its two immediate neighbors). This assessment was performed independently for the medial and lateral compartments, yielding three distinct metrics for analysis: the mean medial JSW, the mean lateral JSW, and the mean JSW of the joint (the average of the medial and lateral compartments). To ensure reproducibility, all assessments were performed independently by two experienced observers under a double-blind principle; the inter-observer reliability was confirmed to be excellent.

### 2.5. Statistical Analysis

Results were shown as mean ± standard deviation (SD). Associations between R2*-quantified synovial iron and CRP, ESR, DAS28, iron metabolism, joint space width, and bone erosion were analyzed by Spearman’s rank correlation coefficients. Differences between two groups were analyzed using the Mann–Whitney U-test, while differences among multiple groups were analyzed using the one-way ANOVA followed by Tukey’s post hoc tests. Univariate and multivariate ordered logistic regression models were performed to calculate odds ratios (ORs) of the influencing factors, as the degree of bone erosion was assessed using a graded scale. The nomogram was constructed on the results of multivariate analysis, the receiver operating characteristic (ROC) curve and area under the curve (AUC) were used to evaluate nomogram performance. R v4.3.2 (https://www.R-project.org, accessed on 7 May 2025), SPSS v26.0, and GraphPad Prism v8.0 were used in this study. A two-sided *p* < 0.05 was considered statistically significant.

## 3. Results

### 3.1. Demographical and Clinical Characteristics

In total, five patients with OA and six patients with RA were included in the exploratory sample, while 51 RA patients were included in the expanded RA group (Figure 1). In the discovery phase, all patients underwent careful evaluation of iron deposition using both chemical colorimetry and MRI IDEAL-IQ methods. The detailed demographics and clinical characteristics of OA and RA patients are shown in Supplementary Table S2. Compared with OA, RA patients had a longer disease duration and had higher inflammatory markers (ESR and CRP). Among the 51 RA patients who underwent MRI scans, 82.40% were female, with a mean age of 53.88 ± 13.29 years and a mean disease duration of 5.97 ± 6.17 years. Of these, 90.20% were ACPA-positive, and 82.40% were RF-positive (Supplementary Table S3).

### 3.2. Quantitative MRI Assessment in RA Patients

Fifty-one knee joints from RA patients were evaluated for imaging characteristics. The mean synovial R2* was 47.34 ± 16.83 S^−1^, synovial volume was 2309.45 ± 1169.05 mm^3^, mean joint space width was 2.64 ± 1.28 mm, and bone erosion depth was 2.95 ± 1.59 mm. Bone erosion and synovitis were observed in all RA patients. Grade 3 bone erosion was the most frequently observed (43.10%), whereas grades 9 and 10 were absent. Synovitis was most frequently observed at grade 2 (60.80%), while grade 1 was the least frequent (9.80%). BME was most frequently observed at grade 1 (60.80%) and least frequently at grade 3 (2.00%) (Table 1).

### 3.3. MRI R2* Is a Practical Method to Quantify Iron Accumulation in RA Joints

**Figure 3:**
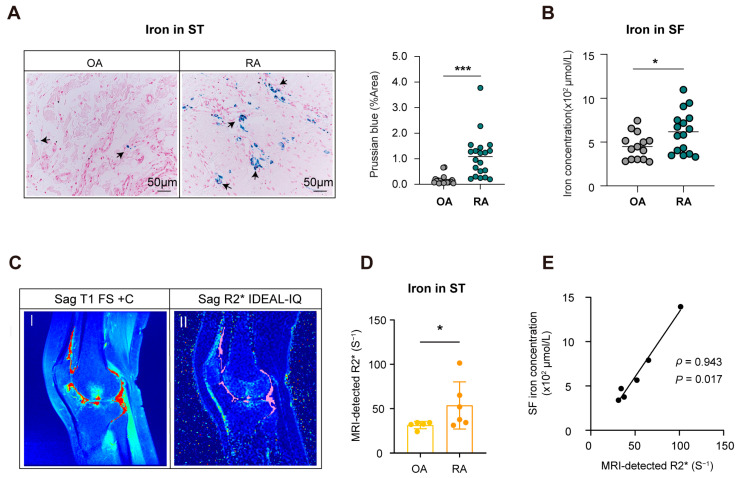
MRI R2* is a practical method to quantify iron accumulation in RA joints. (**A**) The iron deposition of synovium in RA patients stained by Prussian blue (stained blue; indicated by black arrow) against a pink counterstained background, synovium of OA patients was used as a control. Stained areas were more obvious in RA than in OA (*p* < 0.001). (**B**) The iron content in RA-SF was significantly higher than that of the OA group (*p* < 0.05). (**C**) Sagittal MRI images of RA patient showing synovitis on T1 FS + C (I, the ROI in red) and iron level represented by R2* on IDEAL-IQ (II, the ROI in pink). (**D**) R2* in RA ST was significantly higher than that in OA (*p* < 0.05). (**E**) Iron quantification agreed well between chemical colorimetry and MRI IDEAL-IQ (ρ = 0.943, *p* < 0.05) in RA patients. * *p* < 0.05; *** *p* < 0.001. MRI, Magnetic Resonance Imaging; Sag, sagittal; ROI, region of interest; T1 FS + C, T1-weighted fat-suppression contrast-enhanced sequence; IDEAL-IQ, Iterative Decomposition of Water and Fat with Echo Asymmetry and Least-Squares Estimation; SF, synovial fluid; ST, synovial tissues; OA, osteoarthritis; RA, rheumatoid arthritis.

To test whether MRI could accurately represent iron accumulation in knee joints, we compared MRI R2* results with iron detection using traditional methods. Consistent with previous studies, iron deposition in RA synovium was more obvious than that in OA (as indicated by the black arrows in Figure 3A, *p* < 0.001). Similarly, the iron levels in RA-SF were also significantly higher than those from OA patients (*p* < 0.05, as depicted in Figure 3B). To accurately calculate the iron signals in synovium, we outlined the synovial ROI in knee joints from RA and OA patients using the T1 FS + C sequence and recorded the R2* values on IDEAL-IQ sequence (Figure 3C). As expected, R2* of synovial tissues in RA were significantly higher than that in OA, with a mean of 53.66 S^−1^ to 31.38 S^−1^ (*p* < 0.05, Figure 3D). By comparing the iron concentration in SF and the R2* value from the same individual, we found there was a good consistency for iron quantification with chemical colorimetry and MRI IDEAL-IQ examination (*p* < 0.05, Figure 3E). Therefore, MRI R2* could be a practical quantification method for iron deposition in RA joints.

### 3.4. Iron Overload in RA Joint Lesions Correlates with Structural Injury

To explore the pathological role of iron overload in RA progression, we conducted a correlation analysis of focal iron content and the clinical parameters. However, no significant association was found between MRI-detected iron and the circulatory iron metabolic markers, including serum iron, serum ferritin, and transferrin saturation. The systematic inflammatory indicators, such as ESR, CRP, the disease activity score DAS28, and disease duration, were not associated with R2* (Supplementary Table S4). RF and ACPA, as the marker autoantibodies for RA, were not related to R2* either (Supplementary Figure S1). Considering the specific localization of excess iron in joints, we next incorporated the arthritic parameters for further analysis (Figure 3A–C).

**Figure 4:**
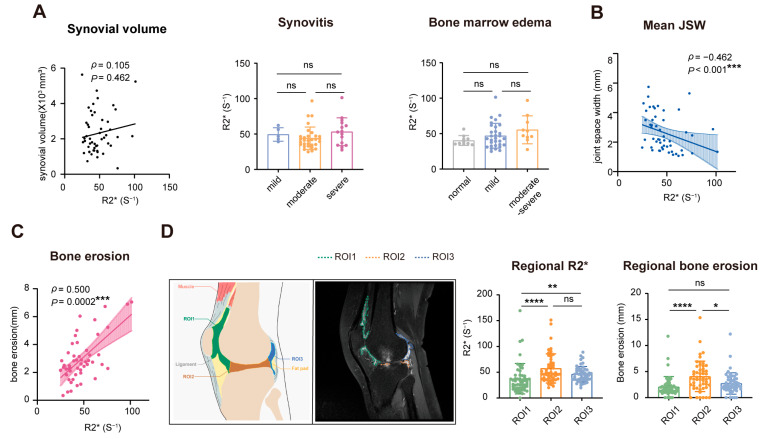
Iron overload in RA joint lesions correlates with structural injury. (**A**) R2* was unrelated to synovial volume, synovitis, or bone marrow edema. (**B**) R2* showed a negative correlation with the mean JSW (mean of the medial and lateral compartments) (ρ = −0.462, *p* < 0.001). (**C**) R2* showed a significant positive association with bone erosion (ρ = 0.500, *p* = 0.0002). (**D**) Iron deposition exhibits distinct distribution patterns across different regions. Hyperplastic synovium in the anterior region, intermediate region, and posterior region was defined as ROI1 (in green), ROI2 (in orange), ROI3 (in blue), respectively. MRI images from another patient to evaluate iron concentrations in synovial membranes across different joint regions on sagittal T1 FS + C images. Iron deposition in joints has unique distribution characteristics: ROI 1 vs. ROI 2, *p* < 0.0001; ROI 1 vs. ROI 3, *p* < 0.01. ROI 2 shows the most extensive bone erosion. * *p* < 0.05; ** *p* < 0.01; *** *p* < 0.001; **** *p* < 0.0001; ns, *p* ≥ 0.05. ROI, region of interest; JSW, joint space width.

Though not associated with synovial volume, synovitis, or bone marrow edema (Figure 4A), the R2* value was significantly correlated with the mean JSW (mean of the medial and lateral compartments) (ρ = −0.462, *p* < 0.001) and bone erosion (ρ = 0.500, *p* = 0.0002) (Figure 4B,C, Supplementary Figure S2). These findings indicated that local iron accumulation could play a role in joint destruction in RA patients.

To enhance the accuracy of focal iron localization and functional assessment, the articular synovium was divided into three compartments on sagittal post-injection T1 FS + C sequences: ROI1 (anterior region), encompassing the prepatellar, suprapatellar, and infrapatellar bursae; ROI2 (intermediate region), representing the central joint space; and ROI3 (posterior region), including the semimembranosus bursa and adjacent posterior recesses (Figure 4D). For each region, synovial iron and bone erosion were quantified with MRI scanning. MRI parameters are shown in Supplementary Table S5. Of the three regions, ROI2 shows the most extensive bone erosion (Figure 4D). Interestingly, iron deposition in joints had unique distribution characteristics, as ROI2 had the highest R2* while ROI1 had the lowest (ROI 1 vs. ROI 2, *p* < 0.0001; ROI 2 vs. ROI 3, *p* > 0.05; ROI 1 vs. ROI 3, *p* < 0.01) (Figure 4D), which is in line with the distribution of bone erosion in knees. Moreover, the correlation between increased R2* and its adjacent bone erosion depth was more pronounced in region 1 (ρ = 0.525, *p* < 0.0001) and region 3 (ρ = 0.494, *p* = 0.0002), but no statistical significance was found in region 2 (ρ = 0.207, *p* = 0.146).

### 3.5. Synovial Iron as an Independent Risk Indicator for Bone Erosion in RA

**Figure 5:**
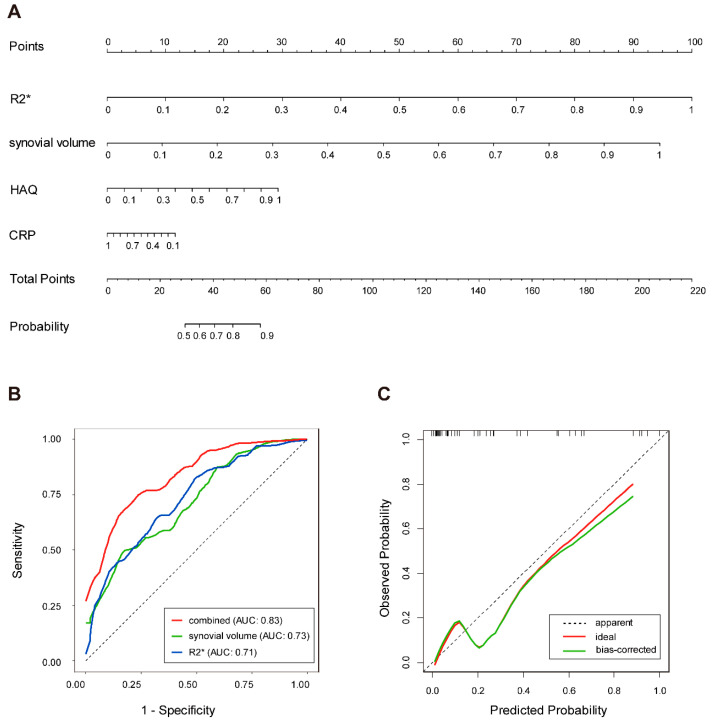
Performance and validation of the nomogram. (**A**) Nomogram for assessing the probability of bone erosion. Instructions: Locate the patient’s value on each variable axis, draw a line upward to determine points earned, sum these points, then draw a line to the probability axis to estimate the likelihood of bone erosion. (**B**) Comparison of receiver operating characteristics curve analysis (ROC) for discriminating bone erosion. The area under the curve was calculated for 3 models as follows: the combined nomogram model (AUC: [0.83]), synovial volume model (AUC: [0.73]), and R2* model (AUC: [0.71]). The diagonal dashed line represents a random classifier (AUC: [0.5]). (**C**) Calibration curve of the nomogram model. AUC, area under the curve.

To further explore the role of synovial iron in bone erosion, all the continuous variables were Min–Max normalized to eliminate different units and magnitudes. The potential factors involved in joint damage were then incorporated into the univariate analysis (Table 2). Ultimately, four factors were selected: R2* (OR = 366.560, 95% CI: 21.396 to 6279.830, *p* < 0.001), synovial volume (OR = 245.904, 95% CI: 13.028 to 4641.389, *p* < 0.001), HAQ (OR = 19.496, 95% CI: 1.113 to 341.625, *p* = 0.042), CRP (OR = 10.707, 95% CI: 1.362 to 84.170, *p* = 0.024) (Table 2). To prevent overfitting, four clinically relevant indicators above were included in the multivariate logistic regression analysis, consistent with the commonly recommended events-per-variable (EPV) ≥ 10 rule of thumb. Multicollinearity was formally evaluated, and all variance inflation factors (VIFs) were <1.5. We found that R2* (OR = 2358.336, 95% CI: 79.306 to 70,130.296, *p* < 0.001) and synovial volume (OR = 1536.134, 95% CI: 46.123 to 511,161.440, *p* < 0.001) were independently associated with the incidence of bone destruction in RA knees (Table 2). Based on these, we developed a nomogram to identify bone erosion and compared the synovial volume model, the R2* model and the combined nomogram model by receiver operating characteristic curve (ROC) analysis (Figure 5A,B). As a result, those three models achieved a high discriminative accuracy in detecting bone erosion in RA knees (Figure 5B). Additionally, the R2* model was quite valid as the correlation between the actual and anticipated findings was strong (Figure 5C). Collectively, synovial iron detected by MRI R2* could serve as an independent factor associated with bone erosion in RA.

## 4. Discussion

In this study, we attempted to quantify articular iron deposition using MRI and assess its contribution to the pathological process of RA. We found that R2* values of synovium acquired through MRI IDEAL-IQ sequence were excellently consistent with chemical iron quantification in synovial fluid and were positively related to the joint space narrowing and bone erosion. These findings suggest that MRI could be a viable and non-invasive method for focal iron assessment in RA, which can be widely applied in clinical practice. Additionally, we identified MRI-detected iron overload in synovial lesions as the independent biomarker associated with bone destruction, implying that early intervention targeting local iron may reverse the disability outcome of RA patients.

Iron metabolism disorder is one of the well-established features of RA. In addition to anemia, which has been commonly correlated with arthritis activity [25], increased iron deposition has been observed in axillary lymph nodes and synovial membrane [11,26]. Consistent with previous studies, our work also identified higher iron levels in RA synovial tissues and synovial fluid compared to OA patients [13,27]. The ectopic deposition of iron in local tissues may result from several factors. Firstly, inflammatory stimuli upregulate hepcidin, a key regulator that inhibits dietary iron absorption in the small intestine and the efflux of intracellular iron, leading to iron redistribution between the circulation and lesions [28]. Additionally, weight-bearing activity or hypoxia may promote arthritic pannus formation and hemosiderin leakage, which further contributes to excessive iron accumulation in tissues [29].

Iron deficiency anemia (IDA) is a common complication of RA [15]; however, the therapeutic effects of iron supplementation in this population remain contentious. Early studies reported exacerbation of synovitis following oral or intravenous iron administration [17], and iron chelation with desferrioxamine was shown to ameliorate soft tissue swelling and bone erosion in Wistar rats [30]. More recently, excess iron in synovial fluid has been implicated in promoting immune imbalance in RA [11], and dietary iron restriction was found to attenuate inflammation in the mouse model of collagen-induced arthritis (CIA) [31]. Despite this preclinical evidence of iron’s detrimental effects at the joint level, recent clinical studies indicated that supplementation with ferric carboxymaltose and intravenous iron saccharate can effectively correct IDA in RA patients without exacerbating disease activity during follow-up [16,32]. Adding to this complexity, longitudinal multi-center cohort studies have demonstrated that greater anemia severity correlates with higher Sharp scores and more significant radiological progression in RA patients [33]. Taken together, these seemingly contradictory findings suggest a compartmentalized role for iron in RA: systemic iron deficiency may contribute to disease severity, while local iron overload within the synovial compartment might be a detrimental factor contributing to arthritis development.

The most widely used methods for assessing articular iron include quantification using atomic absorption spectrophotometry and semi-quantitative evaluation based on Perl’s Prussian blue staining [12,13]. However, both techniques require biopsy specimens, which introduce limitations due to sampling variability and invasiveness. MRI, as a non-invasive alternative, is known to be sensitive to iron due to its ability to disrupt the magnetic field. As early as 2001, Anderson et al. demonstrated a negative correlation between T2* and liver iron concentration (LIC) in non-fibrotic livers, establishing an almost linear positive correlation between R2* (1/T2*) and LIC [34]. Over the years, MRI assessment of liver iron has received FDA approval for clinical diagnosis [35]. Translating this approach to articular applications has required overcoming technical challenges. Initial musculoskeletal studies focused on hemochromatosis arthropathy using T1 relaxation, but this method proved insufficiently sensitive for detecting subtle iron deposition [36,37,38]. In contrast, R2* (1/T2*) and T2* mapping, which are exquisitely sensitive to iron-induced magnetic field inhomogeneities, have overcome this limitation and opened new avenues for articular iron quantification. In our study, we observed that R2* values in the RA synovium were significantly greater than those in OA, consistent with the chemical iron-detection findings in synovial fluid and Perl’s Prussian blue staining in the synovium. Additionally, the strong correlation between MRI-detected R2* values and chemically assessed iron content further validated the reliability of MRI for quantifying focal iron accumulation in RA patients.

MRI is widely used to evaluate focal joint pathology in RA, with the RAMRIS scoring system recommended by OMERACT serving as the standard for hand and wrist joint assessment [24]. However, a standardized MRI scoring approach for the knee has not yet been established. Consequently, previous studies have adopted various methodological strategies, including joint-specific scoring methods [39] as well as adapted applications of RAMRIS for knee assessment [40,41]. In the absence of a validated knee-specific alternative, and given the widespread recognition of RAMRIS for quantifying synovitis, bone marrow edema, and bone erosion in RA, we elected to apply an adapted RAMRIS for knee assessment in this study, focusing on erosive changes at the femorotibial joint.

When exploring the clinical significance of MRI iron detection, we were surprised to find that synovial iron, as represented by R2* value, was not associated with systemic inflammation indicators nor iron metabolic markers in blood. However, it was significantly correlated with adjacent bone erosion. On one hand, this finding reflects the instability and complexity of the circulating indices. On the other hand, it indicates a localized pathogenic process in RA joints, consistent with previous findings that patients achieving clinical remission still exhibit progressive bone erosion on imaging [2]. Mechanistically, excessive iron could promote monocyte-macrophages to osteoclasts [42], and iron-induced ferroptosis of anti-inflammatory macrophages exacerbates local immune dysregulation in arthritic joints. Another interesting finding in this study was the articular spatial variations of iron deposition. Iron quantification of ROI 1 (anterior region), ROI 3 (posterior region) and total synovial area all showed strong association with the progression of joint destruction.

An important overarching point of this study was that MRI-detected synovial iron exhibited strong correlations with bone erosion both in univariate and multivariate logistic regression analyses (*p* < 0.05). The model based on R2* values demonstrated outstanding discrimination and calibration performance. Of note, synovial volume was found to be another significant indicator of bone erosion. Although clinical analysis revealed the relative independence of articular iron and synovial volume, Maria de Sousa et al. have documented that iron stimulates synoviocyte proliferation [43]. Our experimental data also indicated that excess iron exacerbated the invasion and hyperproliferation of fibroblast-like synoviocytes (FLS). Further research about synovial iron and biological characteristics of synovial cells is warranted.

Although this study provided novel insights into the role of synovial iron in RA, several potential limitations should be noted. First, the relatively small sample size of this preliminary investigation may limit the generalizability of the findings; validation in larger, multicenter cohorts is warranted. Second, MRI-based iron assessment was performed exclusively in the knee joint. The knee was selected primarily for methodological reasons, as its larger synovial volume facilitates stable and reproducible MRI-based quantitative evaluation as well as synovial fluid and synovium sampling. However, this joint-specific focus may limit the extrapolation of our findings to other commonly involved joints, such as the small joints of the hands. Third, due to the technical limitation of MRI in distinguishing iron oxidation states (Fe^2+^/Fe^3+^), this study focused on total extracellular iron in synovial fluid supernatant. The specific distribution of iron valence, which may have distinct pathological implications, was not assessed and remains an important avenue for future mechanistic research. Fourth, the use of the 1986 ACR criteria for OA classification, which rely mainly on clinical and radiographic findings, may lead to underdetection of early degenerative changes detectable only by MRI, and future studies incorporating MRI-based assessment could improve the detection of such changes. Finally, the cross-sectional nature of this study precludes the establishment of definitive causal relationships between synovial iron deposition and joint damage. The absence of longitudinal outcomes also prevents evaluation of whether synovial iron accumulation predicts subsequent structural progression. Further prospective, longitudinal studies with larger cohorts and multi-joint assessments are required to elucidate these associations and their potential clinical impact.

## 5. Conclusions

In summary, MRI R2* with IDEAL-IQ sequence is a fast, noninvasive and promising method for synovial iron quantification. As a novel parameter associated with bone erosion, it may help assess the risk of joint disability, so as to facilitate early clinical intervention.

## Figures and Tables

**Table 1:** MRI parameters of RA patients.

	*n*	Value
R2*, S^−1^, mean (S.D.)	51	47.34 (16.83)
Synovial volume, mm^3^, mean (S.D.)	51	2309.45 (1169.05)
Mean Joint space width, mm, mean (S.D.)	51	2.64 (1.28)
Bone erosion, mm, mean (S.D.)	51	2.95 (1.59)
Bone erosion, *n* (%)	51	100.00%
No erosion, scale (%)	0	0 (0.00%)
1–10% of bone eroded, scale (%)	3	1 (5.90%)
11–20% of bone eroded, scale (%)	9	2 (17.60%)
21–30% of bone eroded, scale (%)	22	3 (43.10%)
31–40% of bone eroded, scale (%)	6	4 (11.80%)
41–50% of bone eroded, scale (%)	4	5 (7.80%)
51–60% of bone eroded, scale (%)	3	6 (5.90%)
61–70% of bone eroded, scale (%)	3	7 (5.90%)
71–80% of bone eroded, scale (%)	1	8 (2.00%)
81–100% of bone eroded, scale (%)	0	9–10 (0.00%)
Synovitis, *n* (%)	51	100.00%
No synovitis, scale (%)	0	0 (0.00%)
1–33% of synovitis, scale (%)	5	1 (9.80%)
34–66% of synovitis, scale (%)	31	2 (60.80%)
67–100% of synovitis, scale (%)	15	3 (29.40%)
Bone marrow edema, *n* (%)	41	80.40%
No BME, scale (%)	10	0 (19.60%)
1–33% of BME, scale (%)	31	1 (60.80%)
34–66% of BME, scale (%)	9	2 (17.60%)
67–100% of BME, scale (%)	1	3 (2.00%)

BME, bone marrow edema.

**Table 2:** Logistic regression analysis of factors associated with bone erosion in RA.

Variables	Univariate Regression		Multivariate Regression	
	OR (95% CI)	*p*-Value	OR (95% CI)	*p*-Value
Ages (years)	1.128 (0.129, 9.857)	0.914	-	-
Female	0.294 (0.077, 1.126)	0.074	-	-
BMI (kg/m^2^)	0.437 (0.022, 8.687)	0.587	-	-
Disease duration (years)	4.760 (0.739, 30.661)	0.101	-	-
CRP (mg/L)	10.707 (1.362, 84.170)	0.024	0.405 (0.033, 4.954)	0.479
ESR (mm/1 h)	2.529 (0.477, 13.397)	0.275	-	-
DAS28-ESR	3.286 (0.336, 32.185)	0.307	-	-
DAS28-CRP	3.880 (0.444, 33.927)	0.220	-	-
HAQ	19.496 (1.113, 341.625)	0.042	99.713 (0.530, 178.058)	0.126
R2* (S^−1^)	366.560 (21.396, 6279.830)	*p* < 0.001	2358.336 (79.306, 70,130.290)	*p* < 0.001
Synovial volume (mm^3^)	245.904 (13.028, 4641.389)	*p* < 0.001	1536.134 (46.123, 511,161.440)	*p* < 0.001
Serum iron (μmol/L)	1.153 (0.114, 11.626)	0.904	-	-
Serum ferritin (ng/mL)	16.852 (0.698, 406.747)	0.082	-	-
Transferrin saturation (%)	1.890 (0.107, 33.456)	0.664	-	-
Serum phosphorus (mmol/L)	1.425 (0.072, 28.018)	0.816	-	-
Serum calcium (mmol/L)	88.065 (0.601, 108.296)	0.115	-	-
25 hydroxyvitamin D3 (nmol/L)	1.232 (0.039, 39.196)	0.906	-	-
RF (IU/mL)	4.492 (0.777, 25.982)	0.093	-	-
ACPA (RU/mL)	0.293 (0.016, 5.435)	0.410	-	-

BMI: body mass index; CRP: C-reactive protein; ESR: erythrocyte sedimentation rate; DAS28: disease activity score in 28 joints; HAQ: health assessment questionnaire; RF: rheumatoid factor; ACPA: anticitrullinated protein antibodies.

## Data Availability

The data analysed during the current study are available from the corresponding authors on request.

## Supplementary Materials

The following supporting information can be downloaded at: https://www.mdpi.com/article/10.3390/biomedicines14040749/s1, Figure S1: Synovial iron shows no association with marker antibodies for RA. RF: rheumatoid factor; ACPA: anticitrullinated protein antibodies; Figure S2: Synovial iron was significantly correlated with the medial JSW (ρ = −0.3769, *p* < 0.01) and the lateral JSW(ρ = −0.4380, *p* < 0.01). JSW, joint space width; Table S1: Imaging parameters of MRI sequences; Table S2: Demographics and Clinical characteristics of OA and RA patients for MRI analyse; Table S3: Demographics and Clinical characteristics of RA patients; Table S4: The association between synovial iron and the systemic inflammation and iron metabolic indicators in RA patients; Table S5: MRI parameters in ROI 1-3 of RA patients.

## Abbreviations

The following abbreviations are used in this manuscript:

ACPA	anti-citrullinated protein antibodies
AUC	area under the curve
CRP	C-reactive protein
DAS28	disease activity score in 28 joints
ESGAR	European Society of Gastrointestinal and Abdominal Radiology
ESR	erythrocyte sedimentation rate
FLS	fibroblast-like synoviocytes
GRE	gradient echo
HAQ	health assessment questionnaire
IDEAL-IQ	iterative decomposition of water and fat with the echo asymmetry and least-squares estimation quantitation
LIC	liver iron concentration
MRI	Magnetic Resonance Imaging
OA	osteoarthritis
OR	odds radios
RA	rheumatoid arthritis
RAMRIS	RA MRI scoring system
RF	rheumatoid factor
ROC	receiver operating characteristic
ROI	region of interest
SAR	Society for Abdominal Radiology
SD	standard deviation
SE	spin-echo
SF	Synovial fluid
SJC	swollen joint counts
T1 FS + C	T1-weighted fat suppression contrast-enhanced images
T2-FS	T2-weighted fat suppression
TJC	tender joint counts
VAS	visual analogue scale
