# Supplementary material for: MRI-Based Synovial Iron Quantification Associates with Bone Erosion in Rheumatoid Arthritis
Source: Biomedicines. 2026 Mar 25;14(4):749. doi: 10.3390/biomedicines14040749 (PMC13113699; doi:10.3390/biomedicines14040749)
Supplement: Supplementary file 1 [file biomedicines-14-00749-s001.zip › biomedicines-4165781-supplementary.pdf]

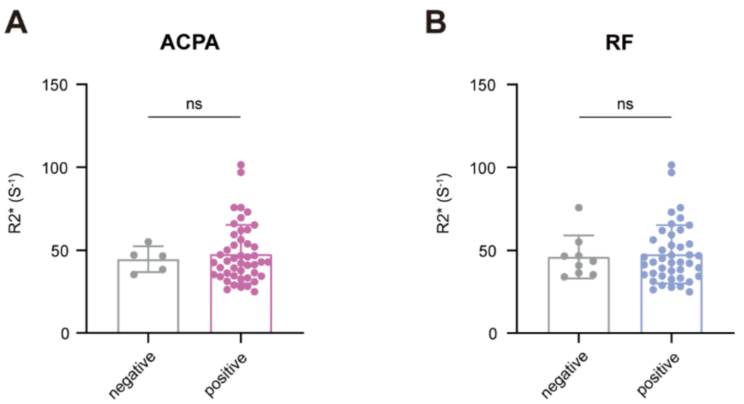

Supplementary Figure S1. Synovial iron shows no association with marker antibodies for RA. RF: rheumatoid factor; ACPA: anticitrullinated protein antibodies.

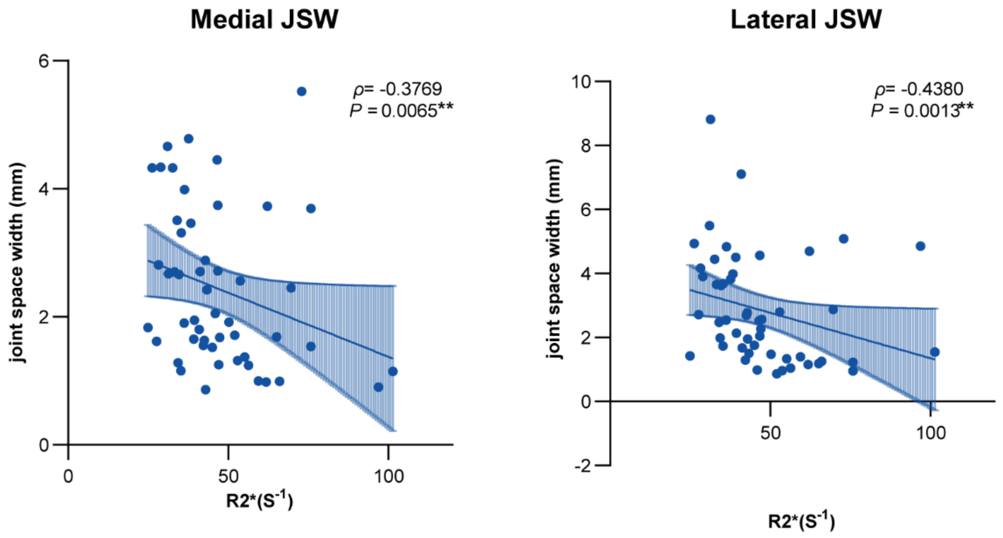

Supplementary Figure S2. Synovial iron was significantly correlated with the medial JSW (ρ = -0.3769, P < 0.01) and the lateral JSW(ρ = -0.4380, P < 0.01). JSW, joint space width.

Supplementary Table S1. Imaging parameters of MRI sequences

Parameters	T1WI	T2-FS	T2-FS	T2-FS	T1FS+C	T1FS+C	T1FS+C	IDEAL-IQ
Plain	Sagittal	Sagittal	Coronal	Axial	Sagittal	Coronal	Axial	Sagittal
No. of section	21	21	21	24	21	21	24	36
Section thickness (mm)	4	4	4	4	4	4	4	4
Section gap (mm)	0.4	0.4	0.4	1	0.4	0.4	1	0
Field of view (mm)	180*180	180*180	180*180	180*180	180*180	180*180	180*180	240*240
Voxel size (mm)	0.6*0.6*4	0.6*0.6*4	0.6*0.6*4	0.6*0.6*4	0.6*0.6*4	0.6*0.6*4	0.6*0.6*4	1.2*1.2*4
Repetition time (msec)	771	3127	3127	5024	607	607	639	13.1
Echo time (msec)	11.2	60	60	60	13	13	14	1.4
Acquisition time	1:16	1:37	1:37	1:37	1:35	1:35	1:40	2:32

T1WI: T1, T1-weighted images; T2-FS: T2-weighted fat suppression; T1 FS +C: T1-weighted fat suppression contrast-enhanced sequence; IDEAL-IQ, Iterative Decomposition of Water and Fat with Echo Asymmetry and Least-Squares Estimation.

Supplementary Table S2. Demographics and Clinical characteristics of OA and RA patients for MRI analyse

	OA (*n*=5)	RA (*n*=6)
Ages, years, mean (*S.D.*)	69.60 (5.94)	54.67 (7.73)
Disease duration, years, mean (*S.D.*)	1.92 (2.86)	4.07 (2.64)
Female patients, *n* (%)	2.00 (40.00%)	3.00 (50.00%)
BMI, kg/m^2, mean (*S.D.*)	23.49 (2.15)	21.84 (2.58)
CRP, mg/L, mean (*S.D.*)	48.54 (40.62)	61.57 (40.62)
ESR, mm/1 hour, mean (*S.D.*)	62.00 (20.16)	94.93 (34.39)
DAS28-CRP	-	6.06 (0.92)
DAS28-ESR	-	6.78 (0.89)
RF-positive, *n* (%)	-	6 (100%)
ACPA-positive, *n* (%)	-	6 (100%)

BMI: body mass index; CRP: C-reactive protein; ESR: erythrocyte sedimentation rate, DAS28: disease activity score in 28 joints; RF: rheumatoid factor; ACPA: anticitrullinated protein antibodies.

Supplementary Table S3. Demographics and Clinical characteristics of RA patients

	RA(*n*)	Value
Demographic and clinical characteristics		
Ages, years, mean (*S.D.*)	51	53.88 (13.29)
Female patients, *n* (%)	51	42.00 (82.40%)
Height, cm, mean (*S.D.*)	51	159.43 (6.97)
Weight, kg, mean (*S.D.*)	51	55.99 (11.33)
BMI, kg/m^2, mean (*S.D.*)	51	22.01 (4.14)
Disease duration, years, mean (*S.D.*)	51	5.97 (6.17)
Tender joint count, mean (*S.D.*)	51	10.33 (7.73)
Swollen joint count, mean (*S.D.*)	51	6.51 (5.15)
Pain VAS, mean (*S.D.*)	51	70.69 (21.49)
HAQ, mean (*S.D.*)	51	1.08 (0.58)
DAS28-ESR, mean (S.D.)	51	6.06 (1.50)
DAS28-CRP, mean (*S.D.*)	51	5.33 (1.45)
Laboratory parameters		
CRP, mg/L, mean (*S.D.*)	51	37.13 (33.73)
ESR, mm/1 hour, mean (*S.D.*)	51	65.96 (35.08)
Serum iron (μmol/L)	51	8.95 (5.34)
Serum ferritin (ng/mL)	51	308.77 (402.93)
Transferrin saturation (%)	51	21.47 (15.39)
Serum phosphorus (mmol/L)	51	1.28 (0.23)
Serum calcium (mmol/L)	51	2.37 (0.17)
25 hydroxyvitamin D3 (nmol/L)	51	58.19 (18.12)
RF-positive, *n* (%)	51	42 (82.40%)
ACPA-positive, *n* (%)	51	46 (90.20%)
Glucocorticoid	51	38 (75.51%)
NSAIDS	51	47 (92.16%)
Tripterygium glycosides	51	7 (13.73%)
csDMARDs		
Methotrexate	51	44 (86.27%)
Azathioprine	51	0 (0.00%)
Leflunomide	51	12 (23.53%)
Sulfasalazine	51	10 (19.61%)
Hydroxychloroquine	51	19 (37.25%)
bDMARDs		

TNF-α inhibitors	51	7 (13.73%)
Etanercept	51	2 (3.92%)
Tocilizumab	51	3 (5.88%)
tsDMARDs		
JAK inhibitors	51	22 (43.14%)

BMI: body mass index; VAS Pain: visual analog scale for pain; HAQ: health assessment questionnaire; CRP: C-reactive protein; ESR: erythrocyte sedimentation rate; DAS28: disease activity score in 28 joints; RF: rheumatoid factor; ACPA: anticitrullinated protein antibodies; NSAIDs: Non-Steroidal Anti-Inflammatory Drugs; csDMARDs: conventional synthetic DMARDs; bDMARDs: biological DMARDs; tsDMARDs: targeted synthetic DMARDs.

Supplementary Table S4. The association between synovial iron and the systemic inflammation and iron metabolic indicators in RA patients

	ρ	*P*-value
CRP (mg/L)	0.007	0.962
ESR (mm/ 1 hour)	-0.010	0.944
DAS28-ESR	0.076	0.597
DAS28-CRP	0.076	0.596
Serum iron (μmol/L)	0.091	0.527
Serum ferritin (ng/mL)	0.128	0.372
Transferrin saturation (%)	0.142	0.321
Synovial volume (mm^3)	0.105	0.462
Disease duration (years)	**0.377**	**0.006**

CRP: C-reactive protein; ESR: erythrocyte sedimentation rate; DAS28: disease activity score in 28 joints; Data is presented as Spearman's rank correlation coefficients.

Supplementary Table S5. MRI parameters in ROI 1-3 of RA patients

MRI parameters in ROI 1-3	*N*	Value
R2* in ROI 1-3	51	-
ROI 1, S^{-1}, mean (*S.D.*)	51	37.38 (29.47)
ROI 2, S^{-1}, mean (*S.D.*)	51	57.33 (28.24)
ROI 3, S^{-1}, mean (*S.D.*)	51	46.13 (14.98)
Bone erosion in ROI 1-3	51	-
ROI 1, S^{-1}, mm, mean (*S.D.*)	51	2.06 (1.99)
ROI 2, S^{-1}, mm, mean (*S.D.*)	51	4.08 (2.93)
ROI 3, S^{-1}, mm, mean (*S.D.*)	51	2.72 (2.06)

ROI, region of interest.
